# Extraction and liposome reconstitution of membrane proteins with their native lipids without the use of detergents

**DOI:** 10.1038/s41598-018-33208-1

**Published:** 2018-10-08

**Authors:** Irina A. Smirnova, Pia Ädelroth, Peter Brzezinski

**Affiliations:** Department of Biochemistry and Biophysics, The Arrhenius Laboratories for Natural Sciences, Stockholm University, SE-106 91, Stockholm, Sweden

## Abstract

Functional studies of membrane-bound channels, transporters or signal transducers require that the protein of interest resides in a membrane that separates two compartments. One approach that is commonly used to prepare these systems is to reconstitute the protein in liposomes. An intermediate step of this method is purification of the protein, which typically involves solubilization of the native membrane using detergent. The use of detergents often results in removal of lipids surrounding the protein, which may alter its structure and function. Here, we have employed a method for isolation of membrane proteins with a disc of their native lipids to develop an approach that allows transfer of the purified membrane protein to liposomes without the use of any detergents.

## Introduction

Removal of membrane proteins from their native membrane environment can destabilize these proteins and/or alter their functional properties^1,2^. Several methods are used to restore native-like conditions. For example, amphipathic polymers (known as “amphipols”) are used to replace the detergent^3^. Lipid nanodiscs, surrounded by membrane scaffolding proteins, are used for both functional and structural studies^4^. Another commonly used method involves reconstitution of purified membrane proteins in lipid vesicles^5^, which allows detailed functional studies in restored native-like conditions. However, all these methods rely on first isolating the membrane protein of interest using detergent. In many cases it is possible to identify and use detergents that yield sufficiently pure, structurally intact and, where relevant, active protein complexes. However, a challenge is to develop a generally applicable method to extract and purify proteins from their native membranes, surrounded by their native lipids, and transfer these discs to a preformed membrane with a well-defined lipid composition.

A path to solving this problem is the use of the amphipathic styrene maleic acid (SMA) co-polymer (for a recent review, see^6^) to extract and isolate the membrane protein of interest together with a disc of native lipids without the use of detergent^7^. The diameter of the native nanodiscs is typically in the range 10–24 nm, where the SMA co-polymer has a thickness of ~1 nm^8^. In recent years this approach has been used to isolate e.g. proton pumps, photosynthetic systems, and ion channels^8–21^. Furthermore, it has been demonstrated that an ion channel, isolated in native nanodiscs, could be transferred to a planar membrane to yield transmembrane ion conduction^14^.

In a recent study we used the SMA co-polymer to extract and purify cytochrome *c* oxidase (Cyt*c*O) from *S. cerevisiae* mitochondria in its native lipid environment using affinity chromatography^11^. The preparation yielded native nanodiscs containing one Cyt*c*O per disc surrounded by ~100 native phospholipid molecules. The Cyt*c*O is a multi-subunit membrane-bound enzyme that catalyzes oxidation of cyt. *c* and reduction of dioxygen to water. The free energy released in this reaction is conserved by linking the electron transfer to proton translocation across the membrane, which results in a proton electrochemical gradient that is used to drive e.g. synthesis of ATP or transmembrane transport processes.

**Figure 1:**
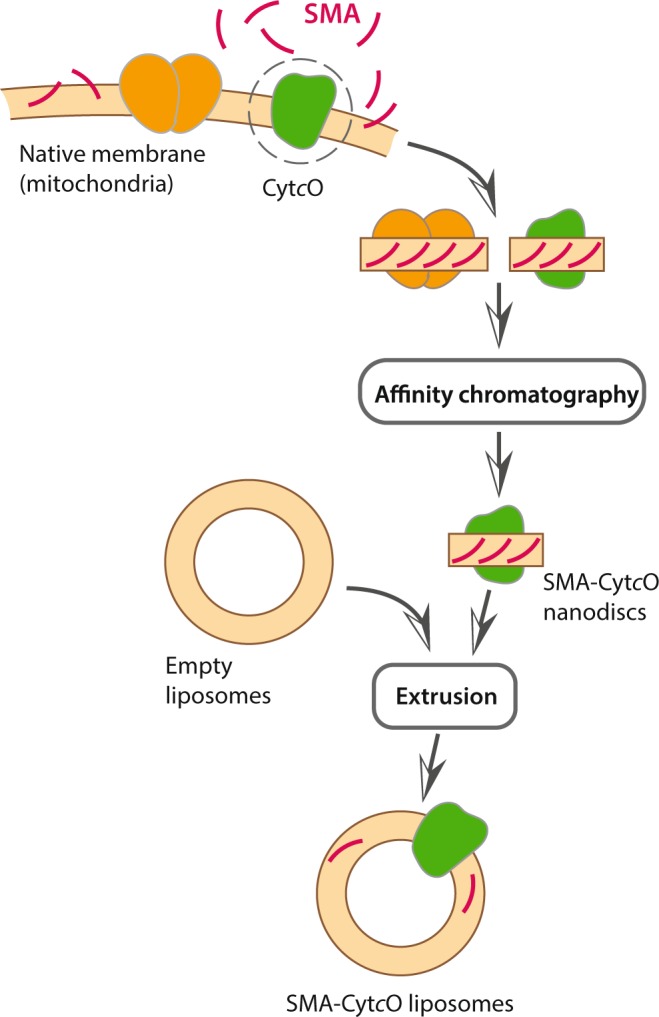
Schematic outline of the method. The SMA polymer (red) is used to extract the protein with its native membrane. The protein in SMA-CytcO native nanodiscs is purified using affinity chromatography. A solution of asolectin is freeze-thawed, which results in formation of empty mono- and multilamellar liposomes^30^. The native nanodiscs containing the protein of interest are mixed with these liposomes after which the sample is immediately extruded through the 100-nm pore filter.

Here, we isolated Cyt*c*O discs with native lipids from *S. cerevisiae* mitochondria using the SMA co-polymer^11^ and incorporated the protein into liposomes with diameters of ~30 nm or ~110 nm without the use of detergent (Fig. 1). The reconstituted Cyt*c*O was active and was shown to generate a proton electrochemical gradient in the order of 100 mV.

## Purification of CytcO in SMA Nanodiscs

*S. cerevisiae* strain MOY764 with a C-terminal His7Strep tag on Cox13^11^ and a strain with a C-terminal His10 tag on Cox6^22^ were used. Mitochondrial membranes were isolated essentially as described in^23^. The SMA co-polymer solution was prepared from the powder form SMA EF30 (Cray Valley) where the ratio of styrene to maleic anhydride was 3:1. It was added to the mitochondrial preparation, non-solubilized membranes were removed at 1.1 × 10^5^ g (40 min), and the SMA-Cyt*c*O native nanodiscs were isolated using affinity chromatography (HisTrap HP, GE healthcare) as described previously^11^. Elution was carried out with 40 mM or 200 mM imidazole for the Cox13-7His and Cox6-10His strains, respectively, where imidazole was removed immediately after the elution. The SMA-CytcO native nanodiscs were stored at 4 °C until use. The position of the His-tag (on Cox6 or Cox13) had no effect on the results obtained in this study.

## Incorporation of SMA-Cyt*c*O into Liposomes

In an earlier study it was noted that nanodiscs containing an ion channel fused spontaneously with pre-formed planar lipid bilayers yielding transmembrane ion transport^14^. Here, we developed two different methods to prepare liposomes and to incorporate the SMA-Cyt*c*O nanodiscs, using sonication or extrusion. These methods of liposome preparation are based on established protocols, but were modified here to exclude the use of detergent.

In all liposome preparations we used L-α-phosphatidylcholine from soybean (type II-S, Sigma, abbreviated in the text as “asolectin”), which was washed as described in^24^.

### Liposomes prepared by sonication

Small liposomes were obtained by sonication of lipids in aqueous solution (see examples in^25–27^). Asolectin (25 mg/ml) was homogenized in 50 mM KH_2_PO_4_-KOH (pH 8.0), 20 mM KCl, purged with nitrogen and sonicated (Vibracell Ultrasonic liquid processor VCX130, Sonics) on ice, without the use of detergent, until the solution became optically clear. The sonication time was 6 min, at 40% amplitude, in pulses of 15 s on and 15 s off. An aliquot of ~10% (by volume) of the SMA-Cyt*c*O sample was added, yielding a final concentration of 0.2–0.4 µM Cyt*c*O. The aim was to obtain maximum of one Cyt*c*O per liposome. The solution was sonicated gently on ice (for 1 min, at 40% amplitude, in pulses of 15 s on and 15 s off). To remove remaining large lipid and titanium (from the sonicator tip) particles, the sample was centrifuged at 1.1 × 10^4^ g for 10 min at 4 °C. The supernatant containing the liposomes was collected, incubated at 4 °C for 1 hour and then kept on ice.

The diameter of sonicated liposomes (without detergent) is typically ~30 nm^28,29^, i.e. the surface area is ~3000 nm^2^. The diameter of each Cyt*c*O native nanodisc was ~12 nm^11^, i.e. their surface area was ~100 nm^2^. Consequently, assuming that the entire content of the disc would be inserted into the liposome, reconstitution of one Cyt*c*Os per liposome resulted in an insignificant increase in the liposome diameter.

### Liposomes prepared by extrusion

Asolectin (20 mg/ml) was homogenized in 50 mM KH_2_PO_4_-KOH (pH 8.0), 20 mM KCl and subjected to six freeze-thaw cycles with freezing in liquid nitrogen and thawing at 35–40 °C in a water bath^30,31^. An aliquot of concentrated SMA-CytcO (about 10% of the total volume) was added to the lipid sample (the final Cyt*c*O concentration was 0.2–0.3 µM). The mixture was immediately extruded at room temperature with a manual mini extruder (Avanti) through a 100-nm filter (Nuclepore, Whatman). After extrusion the liposomes were kept for 20 min at room temperature, then at 4 °C for 1 hour and finally on ice.

The average diameter of the SMA-CytcO-liposomes was found to be ~110 nm, determined using Tunable Resistive Pulse Sensing (TRPS) (qNano, Izon Science, UK): the liposome sample was diluted 200 times (to 1–1.5 nM Cyt*c*O) in a buffer composed of 20 mM HEPES, 200 mM KCl and applied on top of a stretchable pore (NP200). Traces for ~2000 particles were recorded and averaged. The measurement was calibrated with carboxylated polystyrene beads CPC100B (ModeDia 110 nm, Izon).

## Reconstitution Yield for the Extruded Liposomes

To determine the efficiency of Cyt*c*O incorporation into the large (extruded) liposomes, the SMA-Cyt*c*O-liposome suspension was pelleted by centrifugation (1.1 × 10^5^ g, 35 min, 4 °C). Formation of the liposome pellet was clearly observed and the amount of Cyt*c*O in the supernatant corresponded to the amount of non-incorporated SMA-Cyt*c*O (we found that after centrifugation at 1.1 × 10^5^ g for 40 min during the purification procedure the SMA-Cyt*c*O nanodiscs remained in solution, see above). In control experiments we centrifuged the purified SMA-CytcO nanodiscs under the same conditions as was done with the liposomes. These experiments demonstrated a partial sedimentation of nanodiscs yielding a decrease in the concentration of Cyt*c*O in the supernatant by a factor of two (determined spectrophotometrically from the reduced-minus-oxidized absorption spectrum using an absorption coefficient of 24 mM^−1^cm^−1^ at 603 nm^32,33^). It should be noted that the solution density in this control experiment (pure buffer solution) was lower than that during preparation of the SMA Cyt*c*O nanodiscs (excess SMA and lipids). Taking into account the observation of <10% of Cyt*c*O in the supernatant after the liposome centrifugation and the results of the control experiment described above, we estimated that the efficiency of SMA-CytcO incorporation into liposomes was ~85%.

## Orientation of Cyt*c*O in the Membrane

To determine the orientation of the Cyt*c*O in the liposome membrane, the enzyme was first selectively reduced from the outside by 10 µM cyt. *c* and 10 mM sodium ascorbate. The amount of reduced Cyt*c*O was determined from the reduced-minus-oxidized absorption spectrum as described above. Next, sodium dithionite was added until the entire Cyt*c*O population became reduced. The fraction of Cyt*c*O reduced by sodium ascorbate and cyt. *c*, i.e. with the cyt. *c*-binding site accessible from the bulk solution, was estimated to be ~85%. Because a small (~15%) fraction of the SMA-Cyt*c*O native nanodiscs remained in solution (in case of extruded liposomes, see above) and this population was reduced by cyt. *c*, the fraction correctly oriented Cyt*c*O was estimated to be ~70%.

## Estimation of the Membrane Potential Generated by Cyt*c*O

Generation of the membrane potential across the liposome membrane (negative inside) upon Cyt*c*O turnover was monitored using permeant cations: the fluorescent dye TMRE (tetramethylrhodamine ethyl ester, perchlorate, Biotium) or TPP (tetraphenylphosphonium chloride, Aldrich). Formation of the membrane potential upon electron transfer, proton uptake and proton pumping by Cyt*c*O results in accumulation of the positively charged dye TMRE within the liposomes at a locally higher concentration. As a result the TMRE fluorescence is quenched (monitored using a fluorometer, Cary Eclipse, Agilent Technologies).

**Figure 2:**
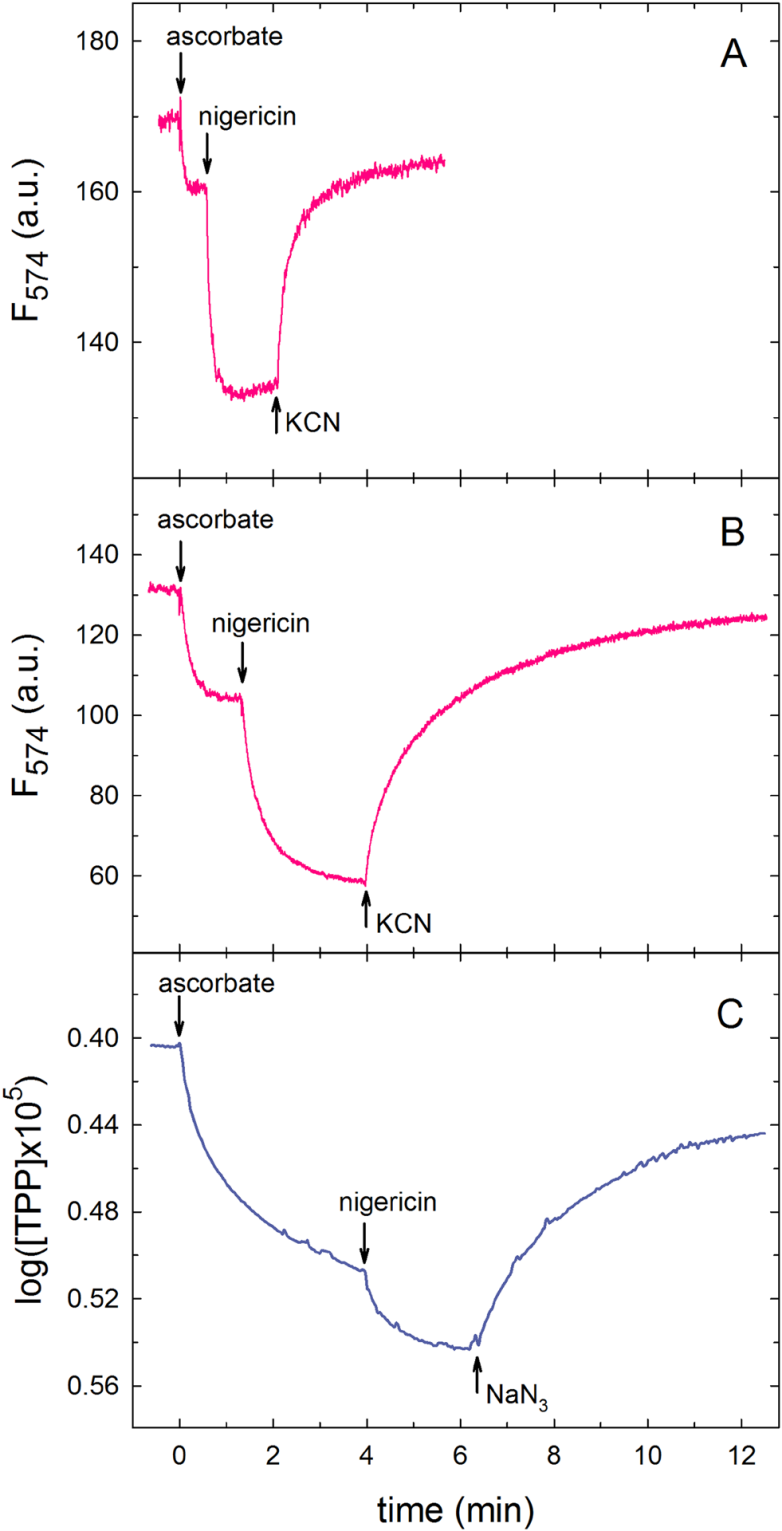
Measurements of membrane potential formed by Cyt*c*O. The membrane potential was monitored by measuring the fluorescence of TMRE at 574 nm (with excitation at 539 nm) with the sonicated (**A**) and extruded (**B**) liposomes. In (**C**) the potential was measured with extruded liposomes using a TPP-selective electrode. In all experiments a solution of SMA-Cyt*c*O-liposomes was added to the assay buffer, supplemented with cyt. *c*. Turnover of Cyt*c*O was initiated upon addition of sodium ascorbate (indicated as zero-time in the graphs), which results in formation of an electrochemical proton gradient across the membrane. The dye (**A,B**) or electrode (**C**) was used to monitor the electrical part of the electrochemical gradient (ΔΨ). The reaction was inhibited by addition of potassium cyanide (**A**,**B**) or sodium azide (**C**). Experimental conditions: 10 mM KCl; 5 mM MgCl_2_, 20 mM KH_2_PO_4_–KOH (pH 8.0), 0.1 mM EDTA (ethylenediaminetetraacetic acid), 10 µM equine cyt. *c*. The additions indicated in the graphs are: 10 mM sodium ascorbate, 0.1 µM nigericin, 2 mM NaN_3_ (**C**)or 2 mM KCN (**A** and **B**). The final Cyt*c*O concentrations were: 8 nM in **A** and **B**, and 12 nM in **C**, respectively. (**A**,**B)**, and 12 nM in (**C**). The traces are presented for the liposomes with SMA-CytcO Cox6-His; similar results were obtained for the liposomes with SMA-CytcO Cox13-His.

As seen in Fig. 2AB, we observed a decrease in fluorescence upon addition of sodium ascorbate in the presence of cyt. *c*. This fluorescence change reflects the electrical part (Δψ) of the electrochemical gradient formed across the membrane. Upon addition of nigericin, an ionophore that equilibrates the potassium and proton concentration gradients, the fluorescence decreased further as the ΔpH component of the gradient was converted into ΔΨ. Upon addition of potassium cyanide, which inhibits Cyt*c*O by binding to the catalytic site, the fluorescence signal increased due to a passive dissipation of Δψ (*via* ion leaks across the membrane). We note that the leak rate was larger with the smaller, sonicated (~30 nm) than with the larger, extruded (~110 nm) liposomes (compare panels A and B in Fig. 2), which presumably reflects a less tight membrane in the more curved liposomes. Because the observed changes in fluorescence could not be calibrated reliably, the approach can only be used to determine the relative values of Δψ and ΔpH.

To quantify changes in membrane potential we measured the redistribution of TPP between the liposomes and the bulk solution using a TPP-selective electrode (Oxygraph-2K, Multisensor system, Oroboros Instruments). Figure 2C shows the data obtained with the extruded liposomes. Upon addition of sodium ascorbate in the presence of cyt. *c* we observed a decrease in the TPP concentration in the bulk solution. The uptake of TPP further increased upon addition of nigericin. From the change in TPP concentration we could estimate the fraction ΔpH to be ~10% of the total electrochemical gradient (see Fig. 2C and equations below). Addition of the Cyt*c*O inhibitor sodium azide caused a release of TPP from the inside of the liposomes into the bulk solution.

For the TPP uptake measurements, Δψ may be estimated from the inside-outside distribution of the cation according to the Nernst equation:

1 $${\rm{\Delta }}{\rm{\Psi }}=2.3\frac{RT}{nF}\,\mathrm{log}(\frac{{[{{\rm{TPP}}}^{+}]}_{{\rm{in}}}}{{[{{\rm{TPP}}}^{+}]}_{{\rm{out}}}})$$

where [TPP^+^]_in_ and [TPP^+^]_out_ are the TPP concentrations inside and outside of the liposomes, respectively, *R* is the universal gas constant, *T* is the temperature; *F* is the Faraday constant and *n* is the number of unit charges transferred, i.e. *n* = 1.

The concentration of TPP inside the liposomes was estimated from the decrease in the TPP concentration in the bulk solution taking into account the internal volume of CytcO-containing liposomes:

2 $${[{{\rm{TPP}}}^{+}]}_{{\rm{in}}}=\frac{{{\rm{\Delta }}[\mathrm{TPP}}^{+}{]}_{{\rm{out}}}\cdot {V}_{{\rm{out}}}}{{v}_{{\rm{lipo}}}\cdot {N}_{{\rm{A}}}\cdot [{\rm{Cyt}}c{\rm{O}}]\cdot {V}_{{\rm{aliquot}}}}$$

where Δ[TPP^+^]_out_ is the decrease in the TPP concentration in the bulk solution upon addition of ascorbate (in the presence of cyt. *c*), *V*_out_ is the volume of the sample, *v*_lipo_ is the volume of a single liposome, *N*_A_ is the Avogadro number, [Cyt*c*O] is the Cyt*c*O concentration in the liposome stock suspension and *V*_aliquot_ is the volume of the liposome stock suspension added to the sample. Typical values were *V*_out_ = 2 ml, *v*_lipo_ = 5·10^−13^ µl, *V*_aliquot_ = 40 µl or 80 µl and [Cyt*c*O] = 0.2 µM. Here, binding of TPP to the membrane was not taken into account (see below).

The fraction of liposomes containing CytcO was estimated from their size and the lipid concentration. It should be noted that the estimation requires that the number of Cyt*c*O molecules does not exceed the number of liposomes (for the extruded liposomes with a diameter of 110 nm the CytcO concentration should be <0.3 µM).

Using Eq. 2 and excluding a fraction of non-incorporated SMA-CytcO, we estimated a Δψ value of 200 ± 20 mV (SD, *n* = 5). As indicated above, in this calculation we did not take into account binding of TPP to the liposome membrane, which could yield an overestimation of [TPP]_in_, i.e. an overestimation of Δψ. We attempted to correct the measured values for this non-specific TPP binding (from the TPP binding at Δψ = 0) with an approach similar to that presented for mitochondria^34^ and obtained 70 ± 10 mV (SD, *n* = 5).

The mechanism for transfer of Cyt*c*O from the SMA native nanodiscs into the liposomes is not known. The SMA is presumably randomly distributed in the lipid bilayer after reconstitution where it could increase the proton permeability of the membrane. An approach to examine this property is to measure the respiratory control ratio (RCR). The parameter is defined as the ratio of the turnover rate for a proton-permeable membrane (in the presence of ionophores valinomycin and FCCP) and for a fully coupled system. We measured RCR values for the SMA-Cyt*c*O in liposomes acquired using both reconstitution methods discussed above. The oxygen-reduction rate was assayed in the presence of 50 µM cyt. *c*, 100 µM TMPD (*N*,*N*,*N’*,*N’*-tetramethyl-*p*-phenylenediamine) and 10 mM ascorbate using an oxygraph (Hansatech). The ionophore concentrations were: 1 µM FCCP (carbonyl cyanide-4-(trifluoromethoxy)phenylhydrazone) and 0.1 µM valinomycin. For the small liposomes (prepared using sonication, see above) the ratio was 1.4 ± 0.2 (*n* = 3), while for the larger liposomes (prepared using extrusion) the ratio was found to be 1.2 ± 0.1 (SD, *n* = 4). It should be mentioned that a presence of non-reconstituted (while enzymatically active) SMA-CytcO yields smaller RCR values. However, we estimated this effect not to be significant. To investigate if a possible presence of SMA in the membrane would influence the RCR values, we tested also reconstitution of the *S. cerevisiae* Cyt*c*O without SMA (purified using detergent based on^23^) using an established protocol, i.e. by sonication of the lipids in the presence of cholate and subsequent dialysis (described in^35,36^). This approach yielded RCR values of 1.3 ± 0.2; *n* = 4, i.e. similar to those obtained upon reconstitution of the SMA native nanodiscs. In other words, the putative presence of SMA has no significant effect on the permeability of the membrane to protons. We note that using the same approach with the bovine heart Cyt*c*O yielded RCRs in the range 7–8. In other words, the relatively small RCRs obtained with the *S. cerevisiae* Cyt*c*O is presumably an inherent property of *S. cerevisiae* enzyme.

## Conclusions

We present a method that allows transfer of a membrane protein with a disc of its native lipids into pre-formed liposomes of well-defined lipid composition and size. Two different methods, employing sonication or extrusion, were used to prepare the liposomes. The proteo-liposomes were impermeable to protons and can be used for studies of protein function, transmembrane transport or structural studies.
